# Corrosion by Polythionic Acid in the Oil and Gas Sector: A Brief Overview

**DOI:** 10.3390/ma16217043

**Published:** 2023-11-05

**Authors:** Mohammadtaghi Vakili, Petr Koutník, Jan Kohout

**Affiliations:** ORLEN UniCRE, a.s., Revoluční 1521/84, 400 01 Ústí nad Labem, Czech Republic; petr.koutnik@unicre.cz (P.K.); jan.kohout@orlenunicre.cz (J.K.)

**Keywords:** polythionic acid, corrosion, corrosion mechanism, mitigation strategies

## Abstract

Polythionic acid (PTA) corrosion is a significant challenge in the refinery industry, leading to equipment degradation, safety risks, and costly maintenance. This paper comprehensively investigates the origin, progression, mechanism, and impact of PTA corrosion on various components within refinery operations. Special attention is afforded to the susceptibility of austenitic stainless steels and nickel-based alloys to PTA corrosion and the key factors influencing its occurrence. Practical strategies and methods for mitigating and preventing PTA corrosion are also explored. This paper underscores the importance of understanding PTA corrosion and implementing proactive measures to safeguard the integrity and efficiency of refinery infrastructure.

## 1. Introduction

Corrosion poses a significant challenge in today’s society, particularly in the industrial sector, where its impact on equipment longevity must be noticed. Recent industrial disasters have resulted in substantial financial losses, highlighting the urgent need to address corrosion-related issues. This concern has prompted petroleum, chemical, and mechanical engineers and chemists to consider the possibility of corrosion occurring in industrial plants thoroughly. It is now recognized that corrosion can influence the chemistry of processes and impact reaction efficiency and product quality. Furthermore, the detrimental effects of corrosion extend beyond financial losses, encompassing large-scale ecological damage [1].

**Figure 1:**
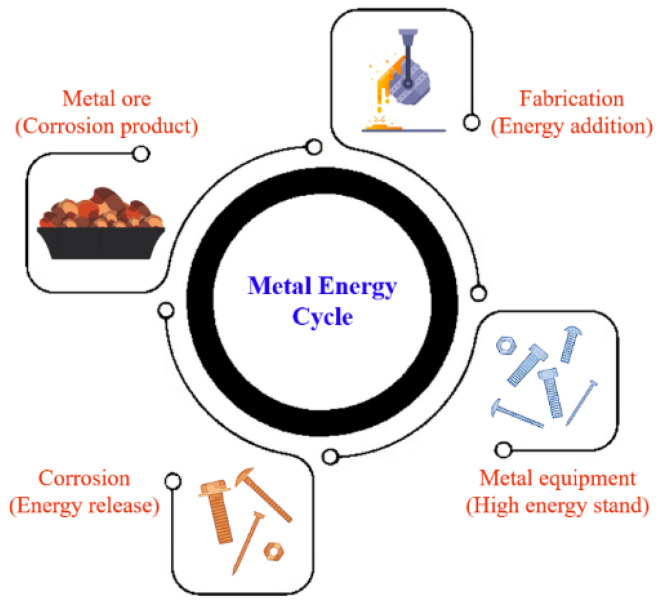
Energy cycle in metal corrosion.

Metal corrosion is an irreversible process driven by the inherent tendency of metals, such as oxides, sulfides, or metal hydroxides, to transition to more stable and lower energy states. Most metals are naturally found in compound form in ores, except for noble metals like gold and platinum, as compounds provide greater thermodynamic stability than their elemental counterparts [2]. The extraction of metals from ores demands a substantial amount of energy to convert them into their pure form. This energy input contributes to the eventual corrosion of the metal when exposed to external elements such as moisture and oxygen (O_2_) (Figure 1). The susceptibility of a metal to corrosion increases with the amount of energy required for its production [3]. Although complete corrosion prevention is impossible, it can be mitigated by slowing down the process. Any factors that promote energy loss from the metal will accelerate corrosion. In industries like oil and gas, corrosion is influenced by various factors, including the presence of water (H_2_O), carbon dioxide (CO_2_), hydrogen sulfide (H_2_S), high temperatures, pressures, and mechanical stresses [4].

Corrosion results from chemical reactions in a corrosive environment, often called electrolytes, which facilitate the transfer of electrons and ions (cations and anions) [5]. These reactions can be categorized into two types: anodic and cathodic. During the anodic reaction, corrosion takes place at the anode. The metal at the anode combines with O_2_ and releases free electrons through oxidation. Typically, the metal with a higher reduction potential is more susceptible to corrosion and is designated as the anode. As a result of oxidation, the anodic metal transforms into its corresponding ion by losing an electron.

On the other hand, reduction occurs at the cathode as the metal accepts electrons from the anode [6]. The corrosion of iron in contact with an electrolyte such as SOO is an example to illustrate the electrochemical reactions. When the electrolyte is slightly acidic, it dissociates into positive hydrogen ions ($H^{+}$) and negative hydroxide ions (${OH}^{-}$). Upon immersing iron in the electrolyte, ionization occurs, causing the iron to dissolve as ferrous ions (Fe^2^⁺). This ionization results from the difference in electric charge at the interface between the solid (iron) and the liquid (electrolyte). The Fe^2^⁺, moving away from the metal surface, undergoes further oxidation, transforming into ferric ions (Fe^3^⁺). These positively charged Fe^3^⁺ are attracted to the negatively charged ${OH}^{-}$ present in the electrolyte, leading to the formation of the corrosion product ferric hydroxide (Fe(OH)_3_) [7].

In the oil and gas industries, corrosion remains a paramount concern due to its profound impact on safety and the substantial economic burdens it imposes on the sector. Aggressive substances, specific flow conditions, and operating temperatures prevalent in oil refineries make corrosion pervasive across numerous processing units [8]. The fluids transported within these industries, often characterized by high sulfur content, contain significant quantities of H_2_S at concentrations ranging from 5 to 5000 ppm [9]. In the refinery where equipment comes into contact with H_2_S-containing fluids, the presence of H_2_O and O_2_ further exacerbates the corrosion potential, creating an environment ripe for corrosive attacks. For instance, a refinery experienced a notable issue involving frequent leaks and the recurrent replacement of components like the nozzle, piping expander, and elbow downstream of the finfan cooler in the sour water stripping unit. Upon thorough examination, it became apparent that corrosion played a substantial role in the observed reduction in wall thickness. This highlights the persistent challenge posed by corrosion in industrial operations [10]. In a separate investigation, a detailed examination of a heat exchanger’s failure on a gas storage platform highlighted the dangers of corrosion in complex equipment. After 24 years of operation, localized galvanic corrosion was discovered on the carbon steel tube sheet, particularly in regions exposed to acidic condensate containing hydrogen sulfide. This corrosive process took place at the interface between the carbon steel and titanium [11]. The H_2_S, O_2_, and H_2_O triad initiate the formation of polythionic acids (PTAs), highly corrosive compounds that wreak havoc on metallic materials. The combination of H_2_S, O_2_, and H_2_O leads to the formation of PTAs, which are highly corrosive and can cause severe degradation of metallic materials, leading to equipment failures, unplanned shutdowns, and substantial economic losses.

In the oil and gas industry, where challenges often arise from processing and handling sulfur-containing materials, understanding PTA corrosion is of paramount importance. This form of corrosion can significantly impact infrastructure and equipment, leading to the thinning of critical components and an elevated risk of structural failure. This, in turn, may result in leaks, ruptures, and, potentially, accidents. Additionally, PTA corrosion diminishes the operational lifespan of equipment, calling for expensive maintenance or replacement. It can also hamper the efficiency of essential processes, leading to reduced output and higher energy consumption. Understanding and reducing the corrosion of PTA in the oil and gas sector has significant practical benefits. It significantly enhances safety by preventing failures and maintaining equipment, reducing the risk of accidents and environmental harm. This approach also extends equipment lifetime, minimizing costly replacements and downtime for repairs. Moreover, it optimizes operational efficiency by ensuring consistent and productive operations, reducing energy consumption, and enhancing flow assurance. Addressing PTA corrosion not only ensures regulatory compliance but also protects the environment, mitigating risks of contamination. Overall, these measures lead to substantial cost savings, improved effectiveness, and a more sustainable and secure operation within the oil and gas industry. An examination of PTA corrosion issues in the oil and gas sectors uncovers several crucial areas warranting further investigation. One such area is the development of advanced coating technologies that provide greater resistance to PTA corrosion. Exploring new materials and adapting them to aggressive environments represents another crucial possibility for investigation. Additionally, a deeper understanding of the electrochemical processes underlying PTA corrosion and the factors influencing its initiation and propagation is essential. Furthermore, emerging technologies such as predictive modeling and real-time corrosion monitoring systems hold great promise in enhancing preventative measures.

These innovative approaches offer the potential to detect early signs of corrosion, enabling timely intervention and mitigation. Moreover, the application of advanced analytics and artificial intelligence in corrosion prediction and management represents a burgeoning field that deserves further exploration. Therefore, this paper aims to not only consolidate existing knowledge but also to shed light on these critical research directions. By identifying these areas of focus and potential technological advancements, it strives to contribute to a more comprehensive understanding of PTA corrosion prevention, ultimately ensuring the long-term integrity and safety of oil and gas infrastructure. This review paper aims to investigate the challenges of PTA corrosion in such sectors and explore effective mitigation strategies. By examining the underlying mechanisms, identifying vulnerable equipment and areas prone to PTA corrosion, and evaluating the efficacy of preventive measures, this review will serve as a valuable resource for industry professionals, researchers, and engineers in developing robust corrosion management strategies. This paper seeks to advance our understanding of PTA corrosion in the oil and gas industries by consolidating existing knowledge, identifying research gaps, and proposing future research directions. Ultimately, the goal is to contribute innovative solutions and best practices to mitigate the detrimental effects of PTA corrosion, ensuring safe and reliable oil and gas infrastructure operation for years to come.

## 2. Polythionic Acids (PTAs)

**Figure 2:**
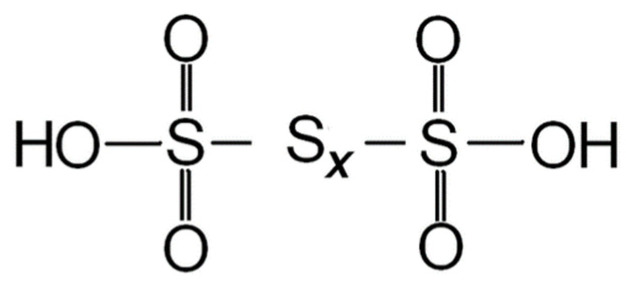
Structure of polythionic acid.

Thionic acids, including PTA, have gained significant attention in the study of crude oil composition and its corrosive properties. Crude oil is a complex mixture comprising a vast array of components, and, among these constituents, thionic acids stand out due to their sulfur and oxygen content. Thionic acids are characterized by sulfonic acid groups (-SO_2_OH), which are directly connected or linked through sulfur atoms (S). The sulfur atoms within the thionic acid compound are solely attached to other S, distinguishing them from other sulfur-containing compounds [12]. As a specific type of thionic acid, PTA exhibits a unique molecular structure characterized by multiple (more than two) sulfur atoms. It is classified as an oxoacid and features an un-branched and linear sulfur chain (-S-S-) attached to an end sulfonic acid group (-SO_3_H), as shown in Figure 2.

The chemical formula H_2_S_x_O_6_ represents PTA, where x signifies the number of sulfur atoms in the chain. While PTA can potentially have an extensive range of x values, exceeding 50 for some compounds, the most commonly encountered PTA falls within the x range of 3 to 6 [13]. Table 1 provides a comprehensive compilation of PTA names based on the number of S found in the chain.

PTA can be synthesized by reacting H₂S and sulfur dioxide (SO_2_) in an aqueous solution. This reaction occurs under typical operating conditions, including ambient pressure, temperature, and a pH range of 3 to 2. When H_2_S is oxidized in the liquid phase, it forms polythionate ions ($S_{x}O_{6}^{2-}$) (Equation (1)). The ratio of H_2_S to SO_2_ in the reactants strongly influences the type and distribution of the resulting products [12]. In the presence of an excess of H_2_S, short-chain polythionates, mainly $S_{4}O_{6}^{2-}$, are preferentially produced. Conversely, when SO_2_ is the dominant reagent, the primary products are longer-chain polythionates with x values ranging from 4 to 8. The pH of the reaction environment also plays a significant role, with higher pH values leading to the formation of shorter polythionates and, ultimately, the generation of thiosulfate ($S_{2}O_{3}^{2-}$) at pH > 8 [13].

(1) $${(2x-5)H}_{2}S+\left(x+5\right){SO}_{2}\to {3S}_{x}O_{6}^{2-}+6H^{+}+(2x-8)H_{2}O$$

PTA exhibits stability only in aqueous solutions and rapidly decomposes at higher concentrations, releasing elemental S, SO_2_, and occasionally sulfuric acid (H_2_SO_4_). Polythionate ions are significantly more stable than their corresponding acids, with PTA containing fewer S in the chain (x = 3, 4, 5, 6) being the most durable. Among these, H_2_S_3_O_6_ is the least stable [14]. The free acid form of PTA slowly decomposes in aqueous solutions, even at room temperature, resulting in the formation of S and sulfates (${SO}_{4}^{2-}$) as end products. The free acid exists solely as a colorless and odorless aqueous solution. In contrast, H_2_S_4_O_6_ is the most stable of the PTAs and exhibits properties similar to H_2_S_2_O_6_ in terms of its heat of neutralization with dilute sodium hydroxide and electrical conductivity [15]. Both H_2_S_5_O_6_ and H_2_S_6_O_6_ acids are relatively stable in acidic solutions but decompose in nearly neutral or alkaline solutions, producing elemental S and lower polythionates ($S_{4}O_{6}^{2-}$ for pentathionic acid and $S_{5}O_{6}^{2-}$ for H_2_S_6_O_6_) [16].

### 2.1. Factors Affecting PTA

Several factors can affect PTA preparation, including (a) the type, concentration, and purity of sulfur-containing compounds used in PTA synthesis and (b) the reaction conditions, such as temperature, pH, and reaction time. Sulfur-containing compounds play a crucial role in preparing PTA as they can impact the final product’s properties, reactivity, and quality. Different sulfur-containing compounds used in PTA development can significantly affect the properties of the prepared PTA, including its concentration, stability, and corrosivity. For example, H_2_S is a highly reactive and corrosive compound that readily reacts with oxygen and water, forming a highly reactive and corrosive PTA solution. PTA can also be synthesized using SO_2_. However, due to the lower corrosivity of SO_2_ compared to H_2_S, the resulting PTA solutions exhibit lower reactivity [17]. Furthermore, the initial ratio of SO_2_ to H_2_S can significantly impact the nature of the reaction products. When there is excess H_2_S, the reaction preferentially produces elemental sulfur and short-chain polythionates, primarily $S_{4}O_{6}^{2-}$. On the other hand, when SO_2_ is the predominant reagent, longer-chain polythionates with x ranging from 4 to 8 are predominantly formed [13]. Strong oxidizing agents, such as hydrogen peroxide or elemental chlorine, convert polythionates into sulfate ions (${SO}_{4}^{2-}$) [18]. This aligns with the fact that polythionates can undergo rapid degradation in the presence of small anions through the breakage of the inner S-S bonds.

Temperature is crucial in PTA synthesis, affecting the reaction rate and product stability. The temperature must be carefully controlled within a generally suitable range of 20 °C to 30 °C to ensure the efficient conversion of the initial materials into PTA [12]. However, at excessively high temperatures, the thermal instability of the compound (e.g., H_2_S and SO_2_) causes the molecular bonds in PTA to become increasingly unstable and weaker, leading to the breakdown of the chemical structure and thermal degradation of the resulting product [19]. The reaction time significantly impacts the synthesis of PTA. Generally, longer reaction times provide sufficient time for the complete conversion of sulfur-containing compounds into polythionate. An adequate reaction time reduces the presence of unreacted compounds and other impurities. Moreover, it helps to prevent side reactions or product decomposition.

In the synthesis of PTA, the pH of the reaction medium plays a crucial role. The sulfur-rich compounds (e.g., H_2_S and SO_2_) used in the preparation of PTA are soluble in H₂O and stable at acidic pH levels. Consequently, PTA can exist and demonstrate improved stability under acidic conditions, typically ranging from pH 2 to 3, where its structure and properties are maintained. Under acidic conditions, the presence of a high concentration of protons ($H^{+}$) facilitates the oxidation of sulfite ions (${SO}_{3}^{2-}$), leading to the formation of $S_{x}O_{6}^{2-}$. The increased protonation rate of these ions stabilizes the prepared PTA molecules and prevents their degradation. Conversely, in a basic environment, the presence of a high concentration of ${OH}^{-}$ results in the formation of ${SO}_{3}^{2-}$, which can further convert into S or ${SO}_{4}^{2-}$. This process leads to the degradation of polythionic acid, making it less stable in strongly basic environments [19].

**Figure 3:**
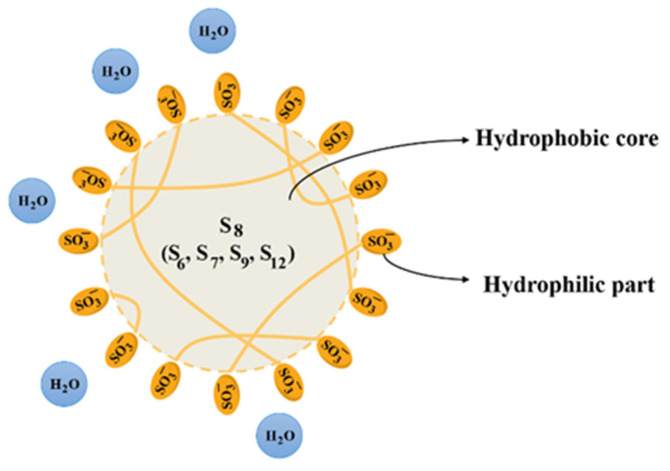
Structure of micelles in hydrophilic micellar colloids.

Polythionate anions exhibit a remarkable property: they tend to form hydrophilic solutions in water, even at high S concentrations. In contrast, elemental sulfur is inherently hydrophobic, and the same applies to sulfur-rich compounds (R-Sx-R) with hydrophobic organic terminal groups. However, strongly hydrophilic groups like SO_3_ can transform these hydrophobic substances into hydrophilic materials. When polythionate anions are dissolved in water, they demonstrate amphiphilic behavior [20]. In the case of higher polythionates, the ions form colloidal structures and aggregate to create ion micelles (Figure 3). On the other hand, short-chain polythionates are soluble in water and do not form micelles. The dispersion can be classified as an S micellar colloid because long-chain $S_{x}O_{6}^{2-}$ undergoes self-organization in water, leading to the formation of micelles. Within these micelles, the hydrophobic sulfur chains are sequestered in the core of the globule. At the same time, the hydrophilic sulfonic acid groups are concentrated on the surface, giving the overall micelle a hydrophilic character. Various factors, such as temperature and pH, can influence the stability of these micelles. However, the content of polythionates and S plays the most significant role: polythionates with lower polythionate and higher sulfur content exhibit lower stability [20].

### 2.2. Polythionates Measurement

Polythionates are challenging to analyze accurately due to their decomposing tendency, particularly in solution. Furthermore, individual polythionate species exhibit similar chemical and physical properties, making their characterization complex. A spectrophotometric method was proposed by Nietzel and De Sesa [21] for determining low concentrations of tetrathionate ($S_{4}O_{6}^{2-}$) ions. This method involves the stoichiometric conversion of $S_{4}O_{6}^{2-}$ to thiocyanate through a reaction with cyanide in an alkaline medium. The excess ferric chloride then forms a red ferric thiocyanate complex. However, this method is unsuitable for measuring higher polythionate concentrations (mainly pentathionate and hexathionate) due to the decomposition experienced by these species. Koh et al. [22] evaluated an alternative photometric method to determine $S_{4}O_{6}^{2-}$ concentrations. This method involves the decomposition of $S_{4}O_{6}^{2-}$ with $S_{2}O_{3}^{2-}\;$and the subsequent measurement of $S_{2}O_{3}^{2-}$ through its decoloration of an iodine solution. However, this method suffers from $SO_{3}^{2-}$ and $S^{2}$ interference and is applicable only within specific pH ranges. Other studies have employed high-speed liquid chromatography, such as the ion exchange column proposed by Wolkoff and Larose [23]. However, the decomposition of the polythionate species hampers the exact determination of polythionates using this method. More recently, ion-pair chromatography has emerged as a modern analysis technique for determining polythionate concentrations in solution. UV detection is utilized in the method proposed by Steudel and Holdt [24], employing ion-pair liquid chromatography with ammonium salts as ion-pair reagents. However, a limitation of this methodology is the significant retention time required for species with more than six S atoms.

## 3. PTA in the Refinery

PTA formation in refineries is commonly observed in units exposed to sulfur-containing compounds like H_2_S and SO_2_, particularly under corrosive conditions involving the presence of O_2_, H_2_O, high temperatures, and low pH [25]. Units such as crude distillation, amine systems, and sour water strippers are particularly susceptible to PTA formation due to the high concentrations of sulfur compounds [26]. The desulfurization processes employed in refineries, including oxidation-extraction desulfurization (OEDS), oxidative desulfurization (ODS), hydrodesulfurization (HDS), adsorptive desulfurization, and bio-desulfurization (BDS), can also contribute to the generation of polythionates as sulfur compounds are converted and transformed during these processes [26]. Table 2 provides a list of refinery units experiencing PTA corrosion issues.

**Figure 4:**
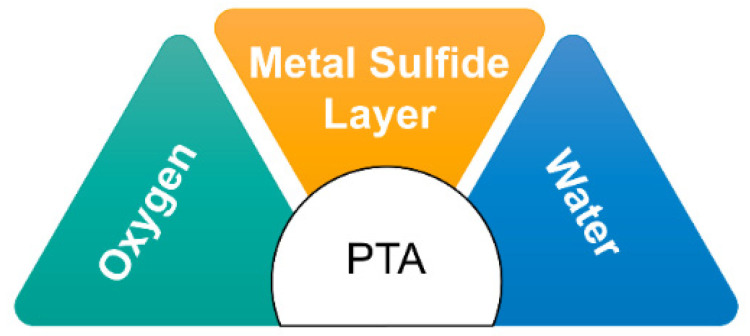
PTA preparation factors.

Operating at high temperatures and pressures, refinery processes can lead to sensitization and reduced ductility in construction materials due to the presence of S and other impurities. The reactions of sulfur impurities with H_2_O and O_2_ result in the formation of H_2_S and SO_2_, which further react to form complex compounds such as $S_{4}O_{6}^{2-}$, polythionates, and polythionic acid [36]. PTA formation primarily occurs in refinery equipment through the reaction of O_2_ and H_2_O with sulfide corrosion products that accumulate on the internal surfaces of the equipment (Figure 4). Moisture, often present from vessel washing or steaming during shutdowns, and oxygen from the air that enters when the vessel is opened contribute to the PTA formation process [37].

While the high operational temperature may cause sensitization of stainless steel (SS), the actual formation of PTA occurs when the material is exposed to air at ambient temperature during shutdown periods [37]. PTA can also be generated by the reaction of H_2_O and O_2_ with oxidizable sulfur species during the combustion of H_2_S in refinery flares. Flare tips, typically composed of austenitic stainless steels (ASS) or high-nickel alloys, are particularly prone to attack by PTA. These acids act as cathodic depolarizers, facilitating metal dissolution at chromium-depleted grain boundaries through cathodic reduction [9].

### 3.1. PTA Corrosion in Refiney

In the field of oil and gas operations, the selection of materials for equipment and infrastructure is a fundamental consideration as their susceptibility to corrosion greatly impacts the lifetime and safety of equipment and infrastructure. Different metals and alloys exhibit varying degrees of vulnerability to corrosion, which significantly impacts the lifetime and safety of equipment and infrastructure. For example, carbon steel, which is widely used in desalination plants, atmospheric desalination, vacuum distillation, catalytic cracking, visebreakers, cokers, and sour water separators, shows corrosion rates between 16 and 315 mpy, depending on the specific environment. It is particularly susceptible to localized pitting corrosion as well as high-temperature oxidation and sulfidation. Cr-Mo steels, present in desalters, atmospheric desalination, and hydrotreating units, have a corrosion rate of around 137 mpy. They may be prone to localized pitting corrosion, hydrogen-induced cracking (hydrogen flaking), and PTA corrosion during the hydrotreating process. Stainless steels of varying grades—316L, 321, and 304L—serve diverse functions in different units, with corrosion rates ranging from 16 to 383 mpy [38]. Their susceptibility to localized pitting corrosion, intergranular cracking, and erosion–corrosion hinges on the specific alloy and environmental conditions. Alloys like Inconel, Monel, and Alloy 800, used in units like catalytic cracking and hydrotreating, exhibit corrosion rates that vary based on the specific alloy composition and prevailing conditions. Copper/nickel alloys, used in atmospheric desalination, record a corrosion rate of about 70 mpy. They are subject to localized pitting corrosion and flow-induced localized corrosion, primarily influenced by factors like naphthenic acid and sulfur. This comprehensive overview of material susceptibility to corrosion in the oil and gas sector highlights the critical importance of selecting the right materials for specific applications, ultimately ensuring the safety and reliability of operations [38]. Table 3 provides a list of materials used in refinery units that face corrosion challenges.

Among the diverse range of metals applied to the oil and gas sector, carbon steel is used as a widely utilized material. However, due to its absence of protective alloying elements like chromium and nickel, it is particularly susceptible to PTA corrosion, especially in environments characterized by high-temperature sulfur compounds. In contrast, stainless steel, endowed with these protective elements, naturally becomes more susceptible to aggressive corrosive agents like PTA [39]. However, some austenitic stainless steels like 304 and 316 can be prone to PTA corrosion due to their higher nickel content and potential sensitization along grain boundaries [40]. Nickel alloys contain a significant amount of nickel, which provides excellent corrosion resistance in a wide range of harsh and corrosive environments. However, specific grades of nickel alloys such as alloy 600 can still experience PTA corrosion [41]. Factors like temperature, pressure, alloy composition, and the chemical environment play significant roles. Higher temperatures and pressures, as well as specific chemical compositions, can make nickel alloys more susceptible. Absolutely, aluminum alloys generally demonstrate better resistance to PTA corrosion than steel alloys thanks to their inherent composition and protective qualities. However, it is worth noting that they can still be influenced by particular aggressive conditions. This susceptibility arises when specific chemicals or compounds come into contact with the protective oxide layer on aluminum, potentially causing localized corrosion. In environments with diverse metals and electrolytes, aluminum may also undergo electrochemical reactions that lead to corrosion. Moreover, under extreme conditions, the protective oxide layer’s effectiveness can be compromised [42].

Components in the oil and gas industry, especially those exposed to elevated temperatures and sulfur compounds, are primarily affected by PTA corrosion. Welded joints, commonly found in pipelines and structural elements, are particularly vulnerable due to alterations in their metallurgical structure during welding. This renders them more prone to corrosion, potentially leading to intergranular or transgranular cracking and compromising structural integrity [43]. Heat exchangers are also notably susceptible to PTA corrosion. This susceptibility arises from the combination of high operating temperatures and potential exposure to sulfur compounds. The manifestation of PTA corrosion in heat exchangers is typically observed as pitting and localized corrosion. These corrosive attacks on the surface of the heat exchanger can significantly damage its heat transfer efficiency, resulting in decreased performance, increased energy consumption, and ultimately necessitating costly maintenance or replacement [44]. Boilers, pressure vessels, and piping systems, experiencing both high temperatures and pressures, are at risk. PTA corrosion in these components leads to localized thinning, which poses safety hazards [45]. Downhole tubing and casings, subjected to harsh downhole conditions, are susceptible to pitting that can weaken their load-bearing capacity. Valves and fittings, especially those in contact with corrosive fluids, face localized corrosion, potentially leading to leaks and reduced operational efficiency.

### 3.2. PTA Corrosion Mechanism

PTA corrosion in refineries occurs in environments containing sulfur-containing compounds such as H_2_S and SO_2_. The mechanism of PTA corrosion involves several stages, including the formation of PTA and polythionates, the attack on metal surfaces, and the acceleration of corrosion. When sulfur-containing compounds in refining processes come into contact with H_2_O, they undergo a series of chemical reactions and form H_2_SO_4_, which can further be oxidized (in the presence of O_2_) to form PTA. Under the influence of oxidizing agents, PTA undergoes oxidation and transforms into polythionate ions such as ${SO}_{4}^{2-}$, which are soluble in water and can become mobile in the refinery environment.

The presence of PTA can lead to localized corrosion of metal surfaces by attacking the protective oxide layers on metal surfaces and initiating corrosion. When carbon steel is exposed to $S_{x}O_{6}^{2-}$, it undergoes immediate attack, releasing hydrogen gas (H_2_) and forming Fe^2^⁺. These ferrous ions react with polythionates to create a protective layer of ferrous sulfate (FeSO_4_) on the metal surface (Equations (2)–(4)). This protective layer is a barrier, safeguarding the metal from further attack. Consequently, the durability of carbon steel tanks and pipes relies on preserving the FeSO_4_ layer. In situations where a protective layer of corrosion products forms together with metal corrosion, the slowest step among the following determines the corrosion rate: the diffusion of the oxidizing agent through the corrosion product layer (FeSO_4_ in the case of carbon steel), the rate of the corrosion reaction itself, or the diffusion rate of the corrosion products away from the metal surface and into the surrounding solution. The corrosion rate is ultimately dictated by the slowest of these steps [46].

(2) $${{2O}_{2}+2H}_{2}O+S_{3}O_{6}^{2-}\to {SO}_{4}^{2-}+2H_{2}{SO}_{4}$$

(3) $${2S}_{4}O_{6}^{2-}+6H_{2}O+{7O}_{2}\to {2SO}_{4}^{2-}+{6H}_{2}{SO}_{4}$$

(4) $$H_{2}{SO}_{4}+Fe\to {FeSO}_{4}+H_{2}$$

### 3.3. PTA Stress Corrosion Cracking (PTASCC)

**Figure 5:**
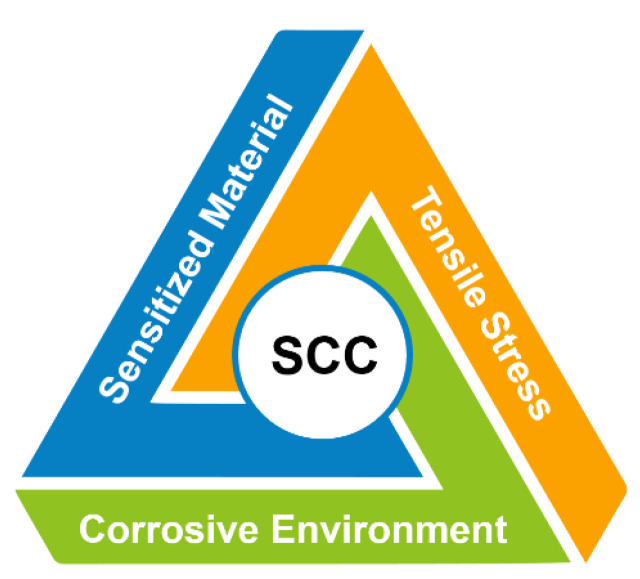
The key factors that contribute to stress corrosion cracking (SCC).

Austenitic stainless alloys are commonly chosen as structural materials due to their favorable mechanical properties and corrosion resistance. However, under certain circumstances, these alloys can experience stress corrosion cracking (SCC) if they have not undergone appropriate fabrication treatments or are exposed to aggressive solution chemistries. SCC refers to the cracking of a material caused by the combined influence of tensile stress and a specific environment. The initiation and propagation of this type of corrosion form are influenced by factors such as sensitized materials (e.g., stainless steel with high carbon content, copper alloys, carbon steels, etc.), the presence of tensile stress (applied, thermal, or residual stress), and specific environmental conditions (e.g., aqueous solutions, moisture, chloride or caustic solutions, high-temperature water, PTA, etc.) [47]. Aggressive ions are required to promote SCC for alloys that develop a protective film. In the case of austenitic stainless steel, PTA and other caustic substances and chlorides can disrupt the protective layer. The contents of Figure 5 illustrate the primary factors responsible for SCC.

**Figure 6:**
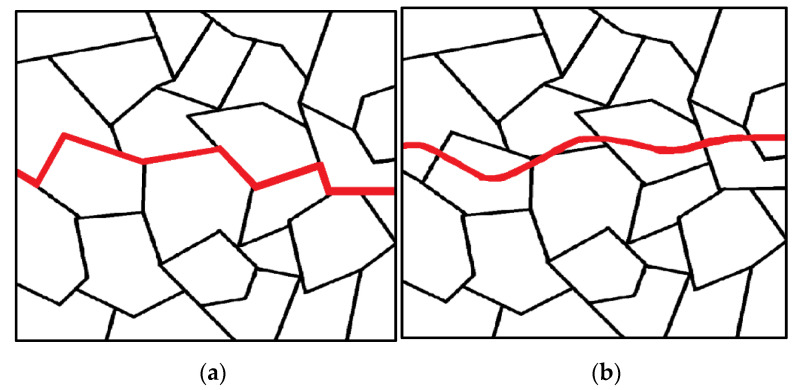
Intergranular fracture along (**a**) and transgranular fracture through (**b**) grain boundaries.

Cracks often originate from corrosion pits and surface imperfections and propagate in a brittle manner. The fracture behavior is not purely mechanical as it is strongly influenced by the corrosive nature of the environment [48]. Once a crack initiates in the metal, it can propagate within the individual grains (transgranular) or along the boundaries between grains (intergranular) (Figure 6). The change in fracture direction occurs when the crack encounters a new grain as the different orientations of atoms within each grain make it easier for the crack to change its path rather than continue tearing through the material [49].

PTA is known to cause stress corrosion cracking (PTASCC) in ASS. PTASCC is a type of material failure typically occurring in areas with high stress or near welds. The challenge posed by PTASCC is prevalent in the oil refining sector, notably in desulfurizer, hydrocracker, and reformer processes. Typically, PTASCC is an issue that occurs internally, affecting the process-exposed side of heater tubes, vessels, or piping. Practically monitoring PTASCC is challenging since the cracking may not manifest until well into a turnaround [45]. The cracking damage is localized and may not be apparent until a leak occurs. PTASCC is particularly severe, with the potential to lead to equipment failures within a day at room temperature. The propagation velocity of PTASCC cracks is faster than that of other forms of stress corrosion cracking. These cracks can propagate rapidly in minutes or hours, leading to containment loss and environmental damage [50]. PTASCC usually happens in austenitic stainless steels and some nickel alloy steels that have been sensitized and develop a sulfide scale on their surfaces when exposed to air and moisture. The cracking occurs during plant shutdowns, subsequent start-ups, or after start-up when the equipment has cooled down. This phenomenon is commonly observed in refinery or petrochemical plants where sulfur contaminants or additives are present to minimize metal-catalyzed coke formation. During shutdowns in sulfide-containing environments, sulfide species (often H_2_S) react with moisture and oxygen to form PTAs that attack the sensitized grain boundaries [51].

PTASCC is influenced by several critical factors, including the surrounding environment, material condition, and applied tensile stress. One essential requirement for PTASCC is the presence of a sensitized microstructure in the alloy characterized by chromium depletion near the grain boundaries. Sensitization typically happens when the metal is exposed to high temperatures ranging from approximately 400 to 800 °C, forming chromium-rich carbides along the grain boundaries. Even austenitic stainless steel grades with low carbon content and stabilization can undergo sensitization. PTASCC primarily manifests as intergranular cracking, propagating along the grain boundaries [52]. Intergranular fracture is commonly observed in metals with a high concentration of brittle particles at grain boundaries. These act as preferential paths for crack propagation, reducing the material’s toughness and damage tolerance. Utilizing processing techniques and heat treatments that prevent the formation of brittle particles at grain boundaries are crucial to guaranteeing strong fracture resistance in structural alloys. In general, PTASCC occurs in sensitized austenitic stainless steels, where a chromium-depleted region adjacent to the grain boundaries exists, accompanied by the precipitation of chromium carbides [45].

A study by Τawancy, H.M. [25] examined heat exchanger tubes from a hydrocracker unit in an oil refinery constructed with 321 stainless steel. Some of these tubes developed cracks at the bent sections after 48 h of operation at 400 °C, following a period of downtime. It was determined that the cracking resulted from stress corrosion induced by PTA. The presence of H_2_S in the environment caused the tubes’ inner surface to transform into a sulfur-bearing scale during operation. This change potentially promoted the formation of PTA by aqueous condensates during downtime, ultimately leading to cracking due to the presence of residual internal stresses. Shayegani and Zakersafaee [53] conducted another investigation into the cracks found in SS347H reactor heater tubes within an isomax unit at a refinery during maintenance. Their research revealed that the reactor tube experienced PTASCC, stemming from the buildup of sulfide scales on its inner surface during operation. When the reactor was exposed to the moisture and atmosphere during maintenance, these sulfide scales interacted with oxygen, forming PTA. This, in turn, led to cracks in the sensitized material of the reactor tube due to the presence of residual internal stresses.

Swaminathan et al. [45] investigated the premature failure of AISI 347 grade fractionator furnace tubes following almost 8 years of operation. It was determined that the failure occurred after shutdown, revealing the presence of carbonaceous residues on the inner surfaces, accompanied by encircling fissures beneath. The potential formation of PTA during the hydrocracker unit shutdown may have played a contributory role. This, in turn, resulted in sensitized alloy 347 tubes experiencing PTASCC. The degradation of the AISI 347 SS furnace tube material was attributed to PTASCC. The material’s sensitized state, likely provoked by localized overheating from coke buildup, exacerbated corrosion induced by PTA formation during the shutdown. Due to existing stresses in the component, the resulting pits acted as stress amplifiers, setting off and propagating intergranular cracking in the circumferential direction.

Khalifeh et al. [54] also observed a similar occurrence, where the tube sheet adjacent to the weld joint became sensitized due to excessive heating from damp carbonaceous deposits. These deposits were notably rich in caustic and chlorides. Subsequently, cracks were exacerbated by the presence of PTA. The accumulation of deposits on the tube sheet was attributed to errors during shutdowns. The overheating of AISI 316 L materials resulted from the insulating effect of these deposits. Sensitization of the material arose as a consequence of this overheating.

Additionally, the combination of sulfur in the process gas and moisture led to the formation of PTA during shutdowns. The coexistence of residual stresses induced by rigorous machining and welding and operational thermal stress contributed to developing the necessary tensile stress for PTASCC. Failures were further aided by the leakage of concentrated water and aggressive agents like caustic and chlorides through the cracks. Upon considering all failure aspects, it was concluded that the material was sensitized, likely due to excessive heating at the deposit sites. This sensitized material ultimately failed due to SCC induced by PTA, with the presence of chlorides and caustic, exacerbating the failures.

The 347H stainless steel tube in a hydroprocessing furnace developed a crack shortly after shutdown, with exposure to sulfur compounds on both its internal and external surfaces [55]. It was determined that the crack initiation occurred on the external surface. Fractography examinations confirmed a brittle fracture, with sulfide corrosion products present. Further observations revealed corrosion within grain boundaries and intergranular cracking, solidifying PTASCC as the primary failure mechanism. The tube’s fire-facing side showed significant sensitization, likely due to extended service or the absence of stabilizing heat treatment during fabrication. Additionally, sulfur compounds in the internal fluid and external environment contributed to PTA formation during shutdown. This, coupled with internal pressure upon start-up, facilitated crack propagation along corroded grain boundaries. This sensitization also notably reduced the ductility of the tube material on the fire-facing side.

### 3.4. PTASCC Mechanism

Understanding the mechanism of PTASCC is essential for applying effective preventive methods. PTASCC is a specific corrosion mechanism in at-risk materials under the combined influence of PTA and tensile stress. PTA concentration, alloy structure, stress level, and environmental conditions can impact the PTASCC mechanism. The PTASCC mechanism involves several steps: (a) formation of PTA through the reaction of O_2_, H_2_O, and sulfur-containing compounds; (b) initiation of the corrosion process by the adsorption of PTA molecules onto the metal surface; (c) application of tensile stress to the metal; (d) initiation of cracking due to the combination of PTA exposure and tensile stress; (e) propagation of cracks; and (f) formation of corrosion products. By understanding these steps, appropriate preventive measures can be implemented to mitigate the risk of PTASCC.

**Figure 7:**
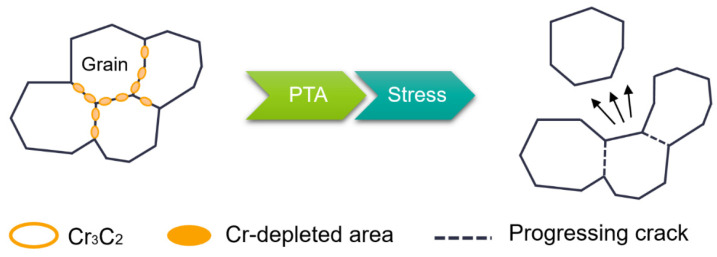
Intergranular cracking caused by PTASCC at grain boundaries.

PTASCC primarily occurs in ASS. The presence of chromium (>10.5 wt%) is crucial for the stainless and austenitic properties of ASS as it enables the formation of a protective surface oxide layer [54]. However, sensitization happens when carbon in the alloy reacts with chromium, leading to the formation of chromium carbides at the grain boundaries. This results in chromium depletion near the grain boundaries, making them vulnerable to corrosive environments, particularly acidic conditions, and causing rapid intergranular cracking. Figure 7 highlights how the corrosive attack of polythionic acid preferentially targets the boundaries between grains, leading to the formation of cracks along these interfaces. When PTA comes into contact with H_2_O, oxy-sulfur anions such as $S_{2}O_{3}^{2-}$ and $S_{4}O_{6}^{4-}$ are produced. These anions can adsorb onto the metal surface and increase the dissolution rate. Dissolution and breakdown of the passive layer lead to the formation of pits, especially in areas with poor passivity or where the protective oxide film has been compromised. At this stage, the combination of residual stresses within the material and operational pressure applied to the pits promotes the formation of initial SCC. These cracks propagate along the grain boundaries, requiring relatively low tensile stress for initiation and growth. ASS with higher carbon grades is more susceptible to PTASCC compared to low-carbon steels (<0.03% C) [56]. Once initiated, the cracks can propagate under electrochemical conditions and tensile stress. Micro-crack presence creates localized high-current-density areas, rapidly dissolving susceptible grain boundaries. The tensile stress from fabrication processes and corrosive environments contributes to the cracks’ further propagation [54].

### 3.5. Detection of PTASCC in Refinery

Detecting PTASCC is most important in preventing risky incidents and ensuring effective maintenance within refinery operations. Regular inspection, non-destructive testing (NDT), and risk-based inspection (RBI) are widely adopted in refineries to identify PTASCC and implement effective prevention and mitigation measures on time. However, each refinery may have specific inspection and detection protocols tailored to industry best practices and regulatory requirements. These practices are essential for safeguarding the integrity of refinery equipment and promoting the safe and efficient operation of the facility. It is worth noting that PTASCC detection is an ongoing process, and a combination of these methods can be employed to ensure comprehensive inspection and continuous monitoring of refinery equipment.

Regular inspection and evaluation of equipment and high-risk areas (e.g., piping, vessels, and tanks) by trained inspectors are crucial for reducing corrosion risks and extending the equipment’s lifespan. This proactive approach involves identifying signs of corrosion, thinning, cracking, and pitting, enabling early detection of damage and preventing equipment shutdowns or disruptions to production processes. Visual inspection, the most widely utilized technique, offers several advantages. It is cost-effective, can be conducted while work is in progress, and allows for the early correction of faults. Furthermore, visual inspection provides valuable insights into incorrect procedures and serves as an early warning system, alerting to developing faults during item usage and enabling proactive measures to avoid complications [57].

NDT techniques are commonly utilized in refineries for the timely detection of PTASCC, ensuring its prevention and mitigation. These methods offer a cost-effective approach to corrosion detection without causing significant operational disruption. Refineries can detect PTASCC without significantly impacting their operations by utilizing these NDT methods, effectively addressing corrosion-related concerns. NDT techniques commonly include infrared thermography, radiography examination, ultrasonic inspection, and eddy current [58].

RBI is a highly effective decision making methodology widely employed for corrosion evaluation, offering invaluable recommendations for mitigating its effects. The primary objective of RBI is to establish an optimal inspection plan that comprehensively and efficiently enhances the safety and reliability of production and processing facilities. This systematic approach integrates inspection data, process conditions, and equipment criticality to prioritize inspection activities effectively. By taking into account factors such as corrosion rates, operating conditions, and historical data, RBI aids in the identification of areas with a higher risk of PTASCC. It is crucial to emphasize that the successful implementation of RBI requires substantial information resources and precise data collection procedures. Moreover, it is important to note that the overall RBI procedure may require significant costs [59].

## 4. Prevention of PTA Corrosion

PTA corrosion failure is a significant concern in petrochemical industries, and preventive measures are crucial to mitigate its occurrence. Efforts are made to prevent the intrusion of O_2_ and H_2_O into the system, which can lead to the formation of PTA. However, several reasons can contribute to their entry, including improper unit shutdowns, O_2_ and H_2_O in steam or wash water used for equipment cleaning, and the inefficiency of catalytic beds in removing O_2_ from the process gas during cracking. Prevention of PTA corrosion is preferable to dealing with cracks after they have already formed. In refineries, effective measures can be taken to control PTA corrosion, such as careful material selection, preventing the entry of O_2_, implementing alkaline washing of surfaces, and avoiding the formation of liquid water. These primary protection methods are crucial in minimizing the risk of PTA corrosion in refinery equipment [60].

### 4.1. Material Selection

Highly alloyed materials are required for effective resistance to different types of corrosion, such as general and PTA corrosion. These materials must have high chromium and nickel content to resist corrosion. Additionally, stabilization with titanium or niobium is necessary to resist intergranular sensitivity and reduce PTA corrosion. Austenitic stainless steel (ASS) is an excellent choice for PTA corrosion. ASS contains high levels of nickel and chromium, which allows the formation of a very thin (1–3 nm) chromium oxide/hydroxide-rich passive film, giving it excellent corrosion resistance [61]. Thus, selecting the appropriate grade of ASS prevents PTA corrosion as the material’s microstructure significantly influences its susceptibility to corrosion. Notably, types 321, 347, and 347LN have shown high resistance against PTA corrosion [62]. For instance, Bradley, S. A. [62] developed a specialized version of type 347LN (with low C, high N, and optimal Nb content). This alloy remains insensitive to long-term exposure to high temperatures, ensuring immunity to PTASCC. With nitrogen infusion, its high-temperature strength aligns with Type 347H austenitic stainless steel, reaching up to 750 °C. It finds suitability in environments ranging from 350 to 750 °C, particularly in areas susceptible to PTASCC, like hydroprocessing heater tubes and reactor circuits, with ongoing sensitization testing underway. A model examination of a hydrocracker heater tube crafted from this alloy, after 100,000 h of operation at an average tube wall temperature of 460 °C, exhibits no signs of sensitization or grain boundary precipitation. This exclusive Type 347LN alloy negates the necessity for downtime protection, resulting in substantial time and cost savings during maintenance periods. The proprietary Type 347LN (in unwelded and weldment forms) revealed no notable susceptibility to intergranular corrosion. Even with a long-term heat treatment of 10 h, there was minimal change in the corrosion rate. Samples subjected to isothermal aging at 565 °C were also assessed for intergranular corrosion. The corrosion rate for the Type 347LN was significantly lower compared to Type 347L or Type 304H. For instance, the un-aged proprietary 347LN displayed a corrosion rate of 17.1 mpy (unwelded) and 11.7 mpy (welded), highlighting its excellent resistance. Even after 1 h at 675 °C, the corrosion rate remained within acceptable limits at 21.3 mpy (unwelded) and 23.7 mpy (welded). As aging time increased, a slight uptick in corrosion rate was observed in the proprietary 347LN alloy, possibly attributed to the formation of sub-micron sigma phase.

On the other hand, materials that are not resistant to PTASCC include some sensitized alloys that are susceptible to the corrosive effects of PTA. This proneness can occur when certain alloys are exposed to specific environmental conditions. The materials composed of austenitic stainless steels, high-nickel alloys, and iron–nickel–chromium alloys are open to attack by PTA. These acids act as cathodic depolarizers, facilitating metal dissolution at chromium-depleted grain boundaries through cathodic reduction [9]. For example, it has been reported that sensitized alloy 600 is vulnerable to rapid SCC even at ambient temperatures when exposed to sulfur-bearing environments such as PTA [41]. The proneness to PTASCC in these materials is primarily due to sensitization, which leads to the precipitation of detrimental phases along grain boundaries. This weakness in the material in those areas makes it more disposed to SCC in the presence of PTA. The application of laser shock peening (LSP) treatment led to a notable decrease in the corrosion rate of the metal alloy. Post LSP treatment, enhancements in corrosion potential and reductions in corrosion current density were observed. Specifically, the corrosion rate decreased by approximately 81%, dropping from 0.26 to 0.05 mm/a for the weld zone. Similarly, for the base metal, there was a decrease of about 61%, with the corrosion rate decreasing from 0.18 to 0.07 mm/a.

It was observed that Undeformed AISI 304, sensitized at 500 °C for 24 h, exhibited a ductile fracture in the PTA solution due to its limited chromium-depleted zone, reducing PTASCC susceptibility [63]. Cold rolling at 20% and 40% before sensitization (at 500 °C for 24 h) made stainless steel prone to PTASCC, which is attributed to severe chromium depletion. Deformation beyond 40% prevented PTASCC despite higher sensitization levels. Only 20% and 40% deformation induced sufficient chromium depletion along grain boundaries for crack propagation. Deformation greater than 40% did not induce this effect, even with a higher degree of sensitization (at 60% deformation).

The importance of pre-sensitization deformation in stainless steel’s susceptibility to SCC is highlighted. This is crucial for engineering components made from stainless steel sheets or plates. Common final operations in the steel industry, like cold rolling, can strain stainless steel to varying degrees. Also, welding can introduce strains up to 20% in the heat-affected zone. When these components are exposed to high temperatures, around 300 °C, for instance, they may undergo severe sensitization, potentially leading to SCC. Deformation may also accelerate SCC by aiding the early growth of pre-existing carbide nuclei, even below the classical sensitization temperature. Moreover, it was found that, when the sample underwent an extended period of holding on the MTS machine followed by gradual straining, it experienced severe SCC. The average crack propagation rate was determined to be 50 nm/s, based on the maximum crack length and the total test duration of the slow strain rate test [63].

### 4.2. Nitrogen Purging

This method involves purging the equipment by displacing the oxygen present in the environment with nitrogen, leading to the generation of an inert environment during shutdown and preventing PTA formation. Moreover, this method can eliminate existing PTA from the metal surface, decreasing maintenance requirements. Additionally, nitrogen’s non-toxicity and non-flammability certify the operational safety and environmental friendliness of this approach. The method is applicable during start-ups, shutdowns, and maintenance processes, proving particularly beneficial for preserving catalysts. Ensuring that the nitrogen used is dry and free of O_2_ is crucial as commercial nitrogen often contains around 1000 ppm of O_2_. When steam is employed for purging, steam injection should be halted before the metal temperature drops to 72 °C (130 °F). Before reaching this temperature threshold, the system should be purged with dry nitrogen [64,65].

### 4.3. Alkaline Washing

The standard method for protecting sensitized stainless steel involves either preventing the formation of PTAs or neutralizing them. To neutralize PTA, washing the equipment with a weak soda ash solution (1–5%) before exposing it to air is recommended. It is essential to soak the equipment for at least 2 h to ensure effective neutralization. Simply spraying the equipment with a soda ash solution is insufficient to prevent PTA formation. If deposits or sludge are present, the solution should be circulated vigorously for at least 2 h [66]. Using a soda ash solution for neutralizing acids should consider the formation of a Na_2_CO_3_ film that can further neutralize acids. It is advisable to assess the influence of alkaline chemicals on catalysts before employing a soda ash wash. Equipment should be hydrojetted with a soda ash solution and reinstalled with the residual soda ash film on surfaces. All equipment surfaces should be thoroughly wetted with the soda wash solution, and a water wash should not follow an alkaline wash. If S-containing fuels have been used in furnace firing, the outside of furnace tubes should be washed with soda ash solution to mitigate the risk of PTA corrosion. It is essential to drain all remaining alkaline wash solutions completely to prevent corrosion caused by carbonates and chlorides through evaporation. Desiccants and dehumidifiers should be used to avoid the formation of liquid water. These operational procedures are designed to prevent the condensation of H_2_O vapor and maintain an alkaline environment by adding ammonia (NH_3_) or Na_2_CO_3_, providing enhanced protection against PTA attacks [64].

### 4.4. Amide Solutions

An alternative approach to washing and neutralizing with an aqueous alkali solution addresses the challenges posed by stress-corrosion cracking due to repulsion by sulfide-containing fluids on the equipment’s surface. Additionally, residual aqueous alkali solution in certain areas can lead to corrosion, making the procedure complex. Instead, washing the equipment with amide solutions prevents the formation of PTA when iron sulfide contacts mineral oil, effectively safeguarding against stress-corrosion cracking of austenitic stainless steel. This technique ensures adequate protection of metal surfaces from PTA-induced corrosion, providing increased durability and dependability for metal equipment exposed to sulfide-containing fluids by leveraging the unique properties of amides and their derivatives to prevent stress-corrosion cracking [67]. Amide corrosion inhibitors possess imide groups with a high electron density, facilitating strong adhesion to the metallic substrate. In this process, the metallic substrate acts as an electrophile, and the imide inhibitors contribute electrons to create a bond with the substrate. The imide structures mainly involve atoms such as oxygen, nitrogen, and sulfur that provide electrons for sharing. Certain amides and derivatives, like thiosemicarbazide, thioacetamide, thiourea, and urea, have demonstrated effective steel inhibitors in acidic solutions. The efficiency and performance of these inhibitors are significantly influenced by their specific structures and the location of the imide group. Remarkably, replacing the oxygen atom in a urea molecule with a sulfur atom (to form thiourea) leads to a remarkable increase in corrosion inhibition efficiency [68].

### 4.5. Dry Air

Eliminating moisture is vital for suppressing corrosion rates in atmospheric conditions. The dry air method effectively limits the risk of polythionic acid (PTA) corrosion by preventing free water formation, a crucial component in PTA production. The dry air method effectively mitigates the risk of PTA corrosion by reducing moisture in the environment to a level where surface wetness cannot form. This preventative technique is essential when metal surfaces are susceptible to corrosion or exposed to sulfur-containing components. Utilizing dry (dehumidified) air offers a cost-effective means to prevent free water formation and reduce the risk of PTASCC. When handling non-regenerable catalysts, which may be pyrophoric, it is essential to keep them moist or isolated from oxygen. Once the catalyst is removed, dry air can be introduced for protection against PTA corrosion. To ensure optimal protection, the dew point temperature of the incoming air should be at least 22 °C (40 °F) lower than the internal surface metal temperature. For example, if the internal metal temperature is 30 °C, the incoming air’s dew point temperature should be 8 °C. Implementing the dry air method aids in maintaining the durability and structural integrity of metal structures and equipment while effectively reducing the risk of PTA corrosion [69].

## 5. Conclusions and Future Prospects

This study serves as a fundamental basis for addressing the corrosion caused by PTA in refineries. The study covers the basic properties of PTA, its role in corrosion, the mechanism of corrosion by PTA in refinery settings, and various protection methods. The key findings from the research are as follows:

- PTA is a thionic acid that contains multiple sulfur atoms, with an unbranched sulfur chain attached to an -SO_3_H group.
- Polythionates can be formed when sulfur-containing compounds such as H_2_S and SO_2_ react in an aqueous solution under ambient pressure, temperature, and variable pH conditions (ranging from 3 to 2).
- PTA is stable only in aqueous solutions and rapidly decomposes at higher concentrations. Among PTAs, those with fewer sulfur atoms in the chain (x = 3, 4, 5, 6) are the most stable, with H_2_S_3_O_6_ and H_2_S_4_O_6_ being the least and most durable PTAs, respectively.
- PTA is soluble in water and exhibits metastability at acidic pH values (ranging from 3 to 2), but polythionates are prone to decomposition under increasing pH and alkali conditions.
- Spectrophotometric, photometric, high-speed liquid chromatography, and ion-pair chromatography methods are suggested for determining polythionate concentration in solution.
- PTA typically forms in refinery equipment when sulfide corrosion products react with O_2_ and H_2_O. They are commonly found on the internal surfaces of equipment, with PTA forming when the material is exposed to air at ambient temperature during shutdowns.
- In the presence of oxidizing agents, PTA can be easily oxidized to form sulfate ions (${SO}_{4}^{2-}$) and H_2_SO_4_, leading to equipment corrosion.
- During refinery shutdowns, PTA can cause stress corrosion cracking in sensitized ASS, which contains chromium (>10.5 wt%) and becomes sensitized through thermal exposure. Precipitation of chromium carbides along the grain boundaries reduces the chromium content in that area, making it susceptible to intergranular corrosion in aggressive environments. Higher carbon grades of stainless steel are more vulnerable to this type of cracking, which is intergranular.
- Primary protection methods in refineries to control PTA corrosion include appropriate material selection, preventing entry of O_2_, alkaline washing of surfaces, and measures to prevent liquid water formation.

More research in PTA corrosion prevention could benefit from focused investigations in several key areas. Firstly, a deeper understanding of the complex interactions between specific environmental factors, material compositions, and corrosion mechanisms is crucial. This could help to refine predictive models and inform targeted prevention strategies. Additionally, research into advanced coatings and surface treatments, tailored for PTA resistance, holds promise. Exploring novel alloy compositions and their performance under varying conditions is another avenue. Furthermore, the development of real-time monitoring and inspection technologies can enhance early detection and response. Incorporating machine learning and AI algorithms for corrosion prediction and prevention is an emerging area with significant potential. Lastly, investigating environmentally friendly inhibitors and coatings aligns with sustainability goals. By delving into these areas, researchers can contribute to more effective and sustainable approaches in PTA corrosion prevention within the oil and gas industry.

Future investigation in PTA corrosion prevention could explore several promising directions. One avenue is the development of smart coatings and protective materials tailored to resist PTA corrosion under specific environmental conditions. Investigating advanced monitoring technologies, such as sensors and non-destructive testing methods, could enable real-time corrosion detection and intervention. Exploring innovative alloy compositions with enhanced resistance to PTA corrosion is another area of interest. Additionally, delving into eco-friendly inhibitors and coatings aligns with sustainability goals in the industry. Furthermore, research on the integration of artificial intelligence and machine learning for predictive corrosion modeling and prevention strategies holds great potential. These avenues collectively offer exciting opportunities to enhance PTA corrosion prevention measures in the oil and gas sector.

## Figures and Tables

**Table 1:** Different polythionic acids (PTAs) and their formulas.

PTA	Trithionic	Tetrathionic	Pentathionic	Hexathionic
**Formula**	$H_{2}S_{3}O_{6}$	$H_{2}S_{4}O_{6}$	$H_{2}S_{5}O_{6}$	$H_{2}S_{6}O_{6}$

**Table 2:** Refinery units affected by PTA corrosion.

Unit/Facility	PTA Formation	Ref.
Hydroprocessing	In units like hydrodesulfurization, hydrocrackers, and hydrotreaters, feedstocks rich in sulfur compounds are processed at high temperatures and pressures. These conditions facilitate the formation of PTA.	[27]
Sour Water Stripping	Sour water stripping units treat the effluent from various units, including delayed cokers, hydrotreaters, hydrocrackers, fluidized catalytic crackers, and visbreaker fractionators, which contain sour water contaminated by phenol, NH_3_, H_2_S, and CO_2_. Under certain conditions, a PTA could be formed.	[28]
Sulfur Recovery	Large quantities of acid gas waste and sour gas streams containing H_2_S are converted to elemental sulfur in these units. If there are leaks or inadequate corrosion protection, PTA can form.	[29]
Amine	These units are used to remove acid gases such as H_2_S and CO_2_ from refinery gas streams and other hydrocarbon streams. PTA formation is possible in these units in case of leakage or insufficient corrosion protection.	[30]
Flare Systems	Flare systems are designed to combust excess hydrocarbons and gases, which may contain chemical substances like SO_x_, CO_2_, and H_2_S. These substances can become corrosive in rainfall, leading to acidic precipitation. If there are leaks or the system is not operating optimally, it could potentially lead to the formation of PTA in areas exposed to oxygen.	[31]
Sulfuric Acid Alkylation	These units play an important role in producing high-octane gasoline and converting isobutane and low-molecular-weight alkenes into alkylates using H_2_SO_4_ as a catalyst. Under certain conditions, a PTA may form.	[32]
Heat Exchangers and Piping Networks	These areas handle materials rich in sulfur compounds and are exposed to specific environmental conditions, including high temperatures and the presence of H_2_O. Therefore, they can potentially be sites for PTA formation under particular conditions.	[33]
Tankage and Storage Facilities	Tankage and storage facilities, particularly those containing and handling sulfur-rich materials and subject to specific environmental factors, have the potential to serve as locations for PTA formation under certain circumstances.	[34]
Sour Water Stripping	Sour water stripping units treat the effluent from various units, including delayed cokers, hydrotreaters, hydrocrackers, fluidized catalytic crackers, and visbreaker fractionators, which contain sour water contaminated by phenol, NH_3_, H_2_S, and CO_2_. Under certain conditions, a PTA could be formed.	[35]
Wastewater Treatment	In these units, water and wastewater contaminated with various chemicals, including sulfur compounds, are processed, and improper management of the condition in these units may lead to the formation of PTA. However, the risk of PTA formation in these units is generally low.	[27]

**Table 3:** Common materials used in refinery units and their corresponding corrosion rates.

Material	Unit/Facility	Corrosion Rate (mpy)
Carbon steel	Desalter	200
Carbon steel, Cr-Mo steels, 12Cr, 316 stainless steel, Monel, and 70–30 copper/nickel alloy	Atmospheric desalination	315
Carbon steel, 9Cr-1 Mo steel, and austenitic stainless steel	Vacuum distillation	417
Carbon steel and stainless steel with refractory lining, Inconel 625, alloy 800	Catalytic cracking	-
Carbon steel, Cr-Mo steels, alloy 825, 321 stainless steel, 347 stainless steel, alloy 800, alloy 800H	Hydrotreating	137
Carbon steel, 316L stainless steel, 405 stainless steel, alloy 825, 9Cr-Al, and graphitized SA 268	Hydrodesulfurization	383
Carbon steel and 2.25Cr 1 Mo steel	Catalytic reforming	48
Carbon steel	Visbreaker	16
Carbon steel	Coker	20
Carbon steel, alloy 400, and Monel 400	Alkylation	100
Carbon steel	Gas treating	10
Carbon steel, 316L stainless steel, alloy 825, Ni-alloy C-276, alloy 2205, alloy 2507, and grade 2 titanium	Sour water stripper	85
Carbon steel, 304L stainless steel, refractory	Sulfur recovery	16

## Data Availability

As a review paper, the information presented in this article is based on the data and findings from the listed source publications in the bibliography.
